# Optimisation of the synthesis of vancomycin-selective molecularly imprinted polymer nanoparticles using automatic photoreactor

**DOI:** 10.1186/1556-276X-9-154

**Published:** 2014-03-31

**Authors:** Kateryna Muzyka, Khalku Karim, Antonio Guerreiro, Alessandro Poma, Sergey Piletsky

**Affiliations:** 1 Kharkiv National University of RadioElectronics, Lenin Ave. 14, Kharkiv 61166, Ukraine; 2 Chemistry Department, College of Science and Engineering, University of Leicester, Leicester LE1 7RH, UK

**Keywords:** Design of experiments, Molecularly imprinted polymer, Nanoparticles, Immuno-like assay, Automatic photoreactor, Optimization, MODDE

## Abstract

A novel optimized protocol for solid-state synthesis of molecularly imprinted polymer nanoparticles (nanoMIPs) with specificity for antibiotic vancomycin is described. The experimental objective was optimization of the synthesis parameters (factors) affecting the yield of obtained nanoparticles which have been synthesized using the first prototype of an automated solid-phase synthesizer. Applications of experimental design (or design of experiments) in optimization of nanoMIP yield were carried out using MODDE 9.0 software. The factors chosen in the model were the amount of functional monomers in the polymerization mixture, irradiation time, temperature during polymerization, and elution temperature. In general, it could be concluded that the irradiation time is the most important and the temperature was the least important factor which influences the yield of nanoparticles. Overall, the response surface methodology proved to be an effective tool in reducing time required for optimization of complex experimental conditions.

## Background

Molecular imprinting, also referred to as template polymerization, is a method of preparation of materials containing recognition sites of predetermined selectivity [1]. Biomimetic assays with molecularly imprinted polymers (MIPs) could be considered as alternatives to traditional immuno-analytical methods based on antibodies. This is due to a unique combination of advantages displayed by MIPs including synthetic procedure that does not require animal inoculation and sacrifice, conjugation of hapten to a carrier protein for stimulated production, the possibility of manufacturing MIPs against toxic substances, excellent physicochemical stability, reusability, ease of storage, and the ability to perform recognition in organic media [2].

The conventional method for preparing MIPs is bulk polymerization [3] followed by grinding and sieving to obtain appropriately sized particles for further use. These are irregular and polydisperse and usually include a large portion of fine particulate material. Extensive sieving and sedimentation are required to achieve a narrow size distribution and to remove fine particles which make this method time consuming and labor intensive. Moreover, the obtained polymers have many limitations, including a high level of nonspecific binding and poor site accessibility for template molecules and therefore are not used in commercial assays.

New methods of MIP synthesis in the form of micro- and nanoparticles offer better control of the quality of binding sites and morphology of the polymer. Micro- and nanostructured imprinted materials possess regular shapes and sizes and a small dimension with extremely high surface-to-volume ratio with binding sites at close proximity to the surface [4]. This greatly improves the mass transfer and binding kinetics. These factors are very important for facilitating binding and improving sensitivity and speed of sensor and assay responses.

Recently, we have developed the first prototype of an automatic machine for solid-phase synthesis of MIP nanoparticles using a reusable molecular template [5]. The instrument for the production of MIP nanoparticles consists of a computer-controlled photoreactor packed with glass beads bearing the immobilized template. It can be suitable (in principle) for industrial manufacturing of MIP nanoparticles. The feeding of monomer mixture, reaction time, and washing and elution of the MIP nanoparticles are under computer control which requires minimal manual intervention. The broad range of parameters which can vary during synthesis of nanoparticles requires extensive optimization of manufacturing protocol. In our work, the composition of monomer mixture is selected using the computational approach developed earlier, which has proven its efficiency and become routinely used in many laboratories worldwide [6]. However, the synthesis of MIPs is a process involving several variables. Its optimization is still a complex task due to the interconnected nature of factors that influence the quality and yield of MIPs [7].

For this reason, the optimization of synthetic conditions by one-variable-at-a-time (OVAT) is unsuitable and cannot guarantee that real optimum will be achieved. The OVAT approach is only valid if the variables to be optimized are totally independent from each other [8]. With the rapidly rising cost of making experiments, it is essential that optimization is done in as few experiments as possible. This is one important reason why statistical experimental design is needed. Design of experiments (DOE) originated as a method to maximize the knowledge gained from experimental data. Compared with conventional methods, multivariate approaches based on DOE allow studying all possible interactions between experimental variables and can significantly reduce the experimental effort needed to investigate the experimental factors and their interactions. These methods are especially valuable for optimization of chemical processes. The examples of application of multivariate DOE include using MODDE 6 software for optimization of supercritical fluid extraction, conditions for the extraction of indole alkaloids from the dried leaves of *Catharanthus roseus*, and GC/MS-based analysis of amino acids and organic acids in rat brain tissue samples [9,10]. Only a few reports discussing the chemometrics approach in rational design of MIPs have appeared. Thus, Kempe and Kempe [11] employed multivariate data analysis (MODDE 6.0 software, Umetrics, Umea, Sweden) for the optimization of monomer and cross-linker ratios in the design of a polymer specific for propranolol. Mijangos et al. [12] used chemometrics (MODDE 6.0 software, Umetrics, Sweden) to optimize several parameters such as concentration of initiator (1,1′-azobis(cyclohexane-1-carbonitrile) and 2,2-dimethoxy-2-phenylacetophenone) and polymerization time required for the design of high-performance MIP for ephedrine.

In the present work, we demonstrate the use of the multivariate DOE approach and MODDE 9.0 software (Umetrics, Sweden) for increasing the yield of MIP nanoparticles synthesized in the automatic photoreactor developed by our team.

## Methods

### Reagents and materials

*N*,*N*′-methylene-*bis*-acrylamide, ethylene glycol methacrylate phosphate, 3-aminopropyltrimethyloxysilane (APTMS), fluorescein *O*-methacrylate, and acetone were purchased from Sigma-Aldrich, Gillingham, UK. Acetonitrile was obtained from Fisher Scientific (Bromborough, UK). *N*,*N*-diethyldithiocarbamic acid benzyl ester was obtained from TCI Europe (Boerenveldseweg 6, 2070 Zwijndrecht, Belgium). Vancomycin was chosen as the model template in solid-phase synthesis of MIP nanoparticles. All chemicals and solvents were of analytical or HPLC grade and were used without further purification.

Phosphate buffered saline (PBS) was prepared from PBS buffer tablets (Sigma-Aldrich, Gillingham, UK) and comprised 0.01 M phosphate buffer, 0.0027 M potassium chloride, and 0.137 M sodium chloride, with pH 7.4, at 25°C. Where necessary, the pH of the buffer was adjusted to pH 7.2 by the addition of HCl.

### Preparation of template-derivatized glass beads

Glass beads (75-μm diameter from Sigma-Aldrich) were activated by boiling in 4 M NaOH for 10 min, then washed with double-distilled water followed by acetone, and dried at 80°C. The beads were then incubated in a 2% *v*/*v* solution of APTMS in toluene overnight, washed with acetone, and subsequently incubated with a 5% *v*/*v* solution of glutaraldehyde, in PBS buffer pH 7.2 for 2 h, after which they were rinsed with double-distilled water. The surface immobilization of the template vancomycin was performed by incubating the beads with a solution of the template in PBS, pH 7.2, overnight at 4°C (concentration of 5 mg mL^-1^). Finally, the glass beads were washed with water and dried under vacuum then stored at 4°C until used. The procedure has been adapted from that published earlier [5].

### Design of the experiment

For the optimization of MIP nanoparticle yield, we have to answer the following questions:

• Which factors have a real influence on yield?

• Which factors have significant interactions (synergies or antagonism)?

• What are the best settings for the photoreactor to achieve maximum output?

• What are the predicted values of responses (results) for given settings of factors?

The experimental design was performed using the software MODDE 9.0 (Umetrics) with central composite on face (CCF) designs with three center points for response surface methodology (RSM) experiments in which the model type is quadratic. The inclusion of center points is usually recommended in DOE since center points give important information on the inherent variability of the experiments, hence allows the estimation of the experimental error of the model. Standard CCF designs use the fractional factorial or full factorial design for a subset of factors in the experiment. RSM was applied to optimize the conditions of MIP nanoparticles preparation using automatic photoreactor with the purpose to maximize the yield of MIP nanoparticles.

A full factorial design with four factors (see Table 1): concentration of functional monomer, irradiation time, temperature of irradiation, and temperature of elution of the low affinity fraction was created, comprising all possible combinations of factor levels. It should be noted that further increasing the number of factors is undesirable due to the proportionally increasing number of experiments required for modeling. Thus, in this work, nineteen initial runs for four factors (*p*) at two levels (*N* = 2^*p*^ + 3 center points) and eight complimentary runs (two runs for each factor) were designed by the software. After excluding 6 runs, where temperature of low affinity waste was smaller than the temperature of irradiation and 2 runs (with similar conditions), the total number of maintained runs was 19. All optimization experiments were performed without replication. The measured response (nanoMIP yield) was calculated from the absorbance spectra intensity measured at wavelength 209 nm, which corresponds to the absorbance maximum of MIP nanoparticles.

**Table 1:** Physical factors studied in present work

**Name**	**Abbreviation**	**Units**	**Settings**
Concentration of monomer	*C*_mon_	%	1 to 5
Irradiation time	*T*_uv_	Min	2.5 to 4.5
Temperature of irradiation	*T*_emp_	°C	10 to 30
Temperature of low affinity waste	*T*__Laf_	°C	10 to 30

The composition of nanoMIP with specificity for vancomycin was adopted from [5]. For clarity purpose, the comparative testing of affinity and specificity of synthesized nanoparticles was outside of the scope of present work. To be sure that the prepared nanoparticles have affinity for the target vancomycin, the particles synthesized in optimum conditions were tested in Biacore experiments (Uppsala, Sweden) with immobilized template as described earlier [4].

### Synthesis of MIP nanoparticles

A generic protocol for the automated synthesis and purification of MIP nanoparticles has been developed and described earlier [5]. The first step involves loading the monomer/initiator mixture, dissolved in a suitable solvent, onto a temperature-controlled column reactor containing the template immobilized onto a solid support. Once the temperature reaches a predetermined set point, polymerization is initiated by UV irradiation of the reactor for the desired reaction time. After polymerization is arrested, the column is washed with fresh solvent at a low temperature. At this stage, unreacted monomers and other low molecular weight materials are eluted along with low-affinity polymer nanoparticles. This leaves the desired high-affinity particles still bound to the phase with immobilized template. These are then collected by increasing the column temperature. Raising the temperature will increase the rate of exchange of the particles with the template phase, reducing the strength of the association, and assist with eluting the particles.

**Figure 1:**
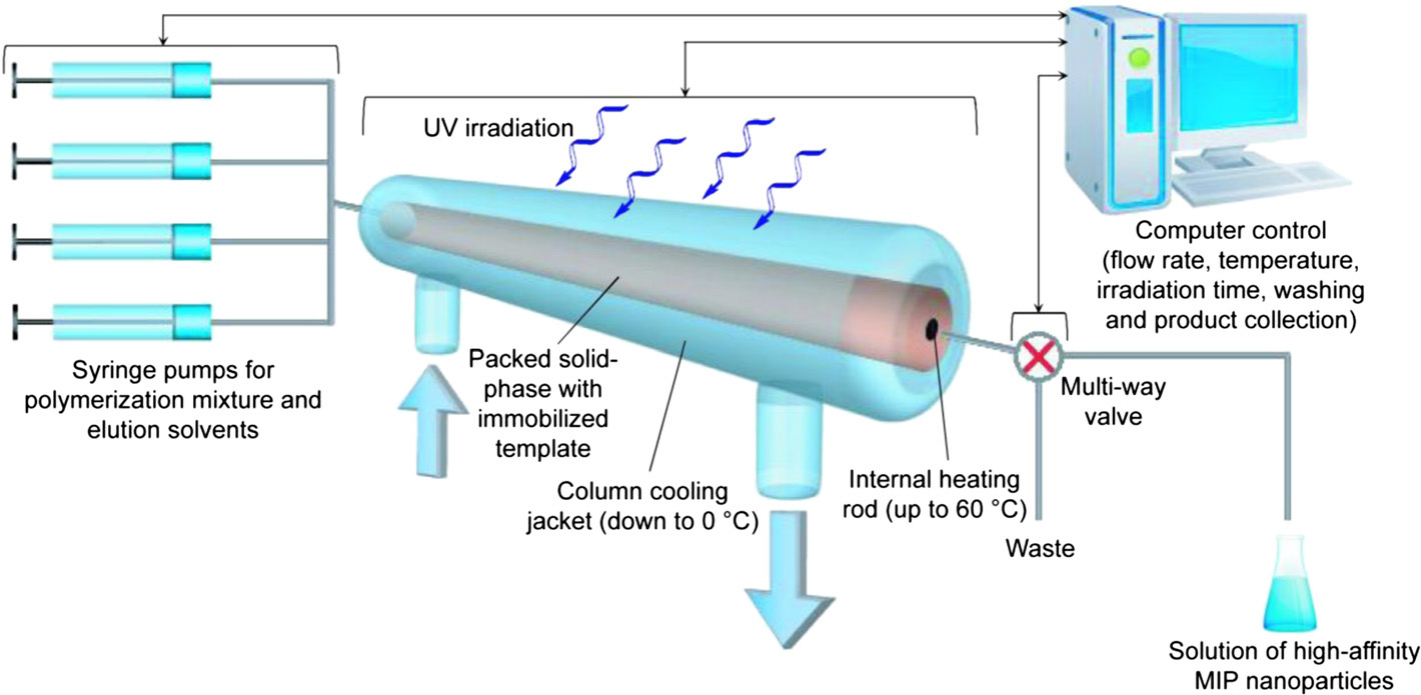
Schematic diagram showing the mode of operation of the automated solid-phase MIP nanoparticle synthesizer.

The experimental setup for the automated synthesis of MIP nanoparticles has been developed with the aim of controlling the column temperature, delivery of the monomer mixture and washing solvents, and UV irradiation time. This comprises a computer-controlled apparatus consisting of a custom-made fluid-jacketed glass reactor with an internal heating element containing immobilized template and connected to pumps which deliver the reaction mixture, wash, and elution solvents. The column is housed in a sealed light box fitted with a UV source that can be activated under software control for a predetermined time to initiate polymerization. The fluid-handling system also employs a multiway valve post-column to direct the high-affinity nanoparticles to a collection vessel or wash solutions to waste (Figure 1).

The benefits of the proposed approach include (i) creation of uniform binding sites, resulting from affinity-based separation on the column; (ii) eliminating contamination of the product with template; (iii) possibility of template reuse; (iv) ease of automation and standardization; (v) the final product is obtained in a pure form free of residues of the template and monomers; and (vi) imprint sites are only formed on one ‘face’ of the particle, allowing post-functionalization of the developed material.

## Results and discussion

The response surfaces generated for this experimental design have been used to verify and calculate the optimum values of significant parameters that influence (increase) the yield of nanoMIPs. The experiments were run in a random order and the yield of nanoparticles calculated from the absorbance values is shown in Table 2. The data shown in Table 2 were analyzed using MODDE 9.0 to generate a model with interaction terms.

**Table 2:** Experimental design matrix used to optimize of MIP nanoparticles yield

**Experiment number**	**Name of experiment**	**Run order**	**Inclusion/Exclusion**	**Concentration of monomer**	**Irradiation time**	**Temperature of irradiation**	**Temperature of low-affinity wash**	**Yield**
1	N1	14	Incl	1	2.5	10	10	3.4
2	N2	19	Incl	5	2.5	10	10	0.796
3	N3	24	Incl	1	4.5	10	10	0.336
4	N4	5	Incl	5	4.5	10	10	0.269
5	N5	26	Excl	1	2.5	30	10	
6	N6	6	Excl	5	2.5	30	10	
7	N7	9	Excl	1	4.5	30	10	
8	N8	4	Excl	5	4.5	30	10	
9	N9	15	Incl	1	2.5	10	30	1.478
10	N10	2	Incl	5	2.5	10	30	0.812
11	N11	13	Incl	1	4.5	10	30	0.739
12	N12	12	Incl	5	4.5	10	30	0.567
13	N13	10	Incl	1	2.5	30	30	0.922
14	N14	22	Incl	5	2.5	30	30	0.937
15	N15	16	Incl	1	4.5	30	30	0.585
16	N16	11	Incl	5	4.5	30	30	0.269
17	N17	23	Incl	1	3.5	20	20	0.75
18	N18	7	Incl	5	3.5	20	20	0.245
19	N19	3	Incl	3	2.5	20	20	1.038
20	N20	8	Incl	3	4.5	20	20	0.488
21	N21	18	Incl	3	3.5	10	20	0.833
22	N22	20	Excl	3	3.5	30	20	
23	N23	17	Excl	3	3.5	20	10	
24	N24	25	Incl	3	3.5	20	30	1.768
25	N25	27	Incl	3	3.5	20	20	0.858
26	N26	21	Excl	3	3.5	20	20	
27	N27	1	Excl	3	3.5	20	20	

**Figure 2:**
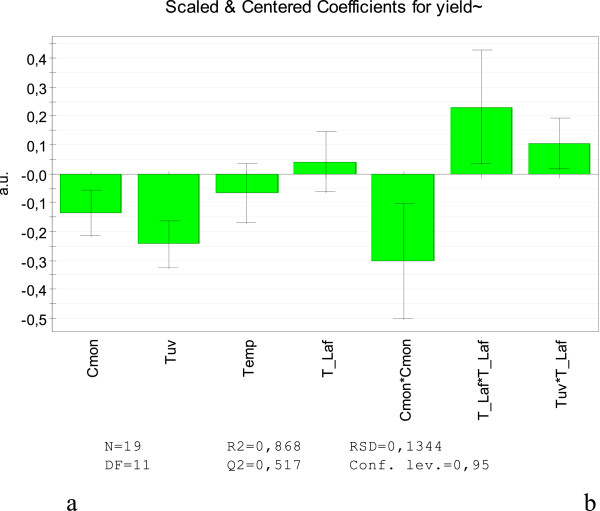
**A graphical representation of the coefficients of the models after trimming a small and not significant terms.***C*_mon_, concentration of monomer; *T*_uv_, irradiation time; *T*_emp_, temperature of irradiation; *T*__Laf_, temperature of low affinity waste.

**Figure 3:**
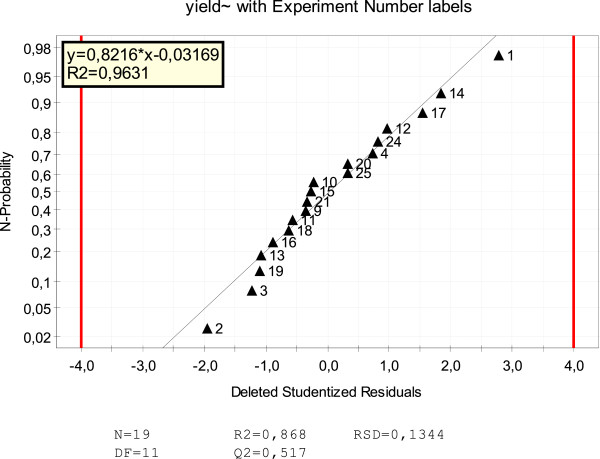
The residuals of a response vs. the normal probability of the distribution.

The quality of the model is R2 = 0.868, Q2 = 0.517 (Figure 2), where R2 is the goodness of fit value and is a measure of how well the model fits to raw data, and Q2 is goodness of prediction and estimates the predictive power of the model. Reproducibility is a measure of the variations of the response. The quality of the model has also been confirmed by the fact that the points on the normal probability plot (Figure 3) show a nearly linear pattern, which indicates the normal distribution. Bar charts provide an overview of which factors most influence MIP nanoparticles’ yield. The results presented in Figure 2 allow the conclusion that the concentration of monomer and the time of irradiation have the biggest effect on the output.

**Figure 4:**
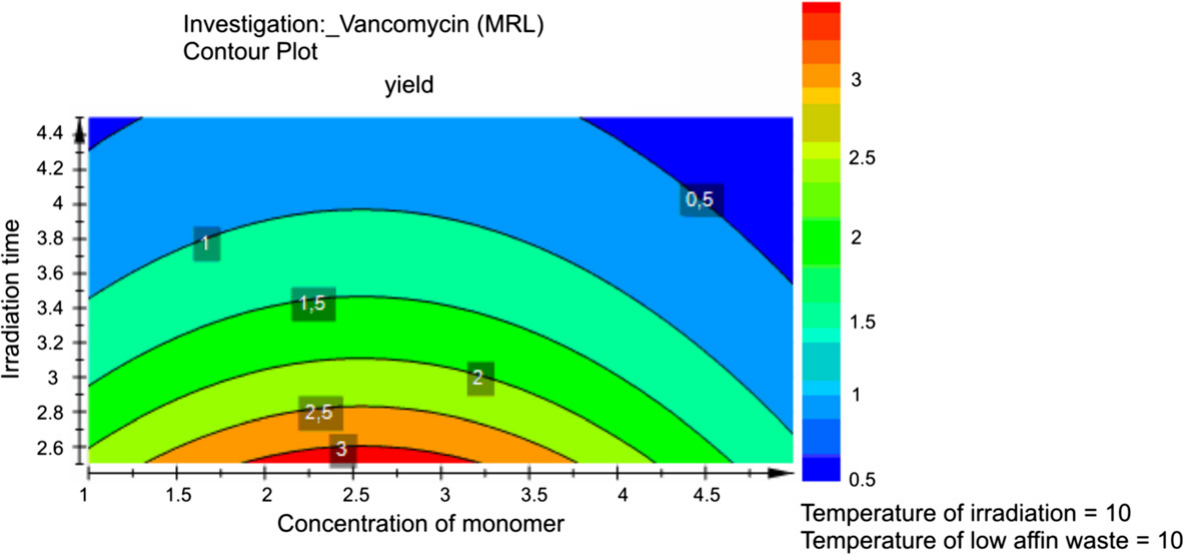
Contour plot of the yield of MIP nanoparticles.

Finally, graphically, the model is visualized by drawing 2D contour plots (Figure 4). From such plots the best or optimal conditions are derived. A 2D contour plot, which has been obtained in this work, shows the isoresponse lines (MIP nanoparticles yield) as a function of the levels of two factors (irradiation time and concentration of monomer) that can change and two factors which should be constant (temperature during UV irradiation and temperature of low-affinity MIP nanoparticles waste). It must be noted that all these factors might also affect the quality and quantity of MIP recognition sites. Therefore, from analysis of Figure 4, it can be concluded as follows:

i. The maximum level of anti-vancomycin nanoMIPs yield is equal to 3.4 a.u., which corresponded to the range of functional monomer concentration between 1.8% and 3.25% (percentage ratio of functional monomer in polymerization mixture). The decrease of monomer concentration to the minimum setting in this work value (1%) or increase to the highest possible (5%) has not led to a significant reduction of response (2 a.u.). The influence of the percentage ratio of functional monomer in the polymerization mixture on the response can be explained by the fact that the ratio of functional monomer to cross-linker affects the rigidity of the polymer matrix. This in turn affects an association degree of the polymerization mixture with the immobilized template (vancomycin) and consequently affects the quantity of nanoMIP with low affinity, which should be washed out during the first elution. Therefore, theoretically, the yield of high-affinity particles obtained during the second elution will decrease with increasing amounts of low-affinity particles produced during the first elution and vice versa.

ii. The yield of nanoparticles depends on the irradiation time in the entire range of values tested in this work. The maximum yield (3.4 a.u.) was observed at 2.5 min of UV polymerization. Further increase of irradiation time from this point has led to a significant reduction of the response, which reached a minimum (0.5 a.u.) at the irradiation time of 3.4 min. It is reasonable to assume that a prolonged polymerization time increases the diameter of particles which are less efficient in binding to the immobilized template due to sterical factors. Therefore, it can be concluded that a polymerization time of 2.5 min is optimal for the production of nanoMIPs with good binding properties.

iii. Temperature equal to 10°C was the lowest value (used in this work and predicted by RSM as theoretical optimum) of the temperature during UV irradiation. Moreover, theory and our previous investigation [5] indicated that the requirement for using low temperatures is best met by initiating the polymerization reaction through photochemical means, since it can be performed at or below room temperature.

iv. Temperature of 10°C was the minimum value for the wash of low-affinity MIP nanoparticles set in this work. This temperature has been found optimal for removal of nonspecific nanoMIPs [5].

It should be noted that the binding properties of the synthesized (under optimal conditions) anti-vancomycin MIP nanoparticles were analyzed by SPR experiments (Biacore) using chips with immobilized templates as described earlier [5]. The apparent dissociation constants xcalculated for vancomycin nanoMIPs was *K*_*d*_ = 3.4 × 10^-9^ M. This result has proven that by using automatic solid-phase synthesis under optimized parameters, it is possible to produce high-quality MIP nanoparticles which resemble, in practical terms, monoclonal antibodies.

## Conclusions

In this study, a DOE approach (the software MODDE 9) was employed to evaluate the influence of concentration of functional monomer in the polymerization mixture, time and temperature of UV irradiation, as well as temperature of elution of the low-affinity fraction on the yield of MIP nanoparticles which have been produced by the automatic photoreactor developed by our team. The use of RSM significantly reduced the experimental efforts needed to investigate factors and their interactions. The applications described in this paper clearly show the practical usefulness of experimental design for the optimization of synthetic protocol, in particular complex experimental conditions.

Thus, the yield of MIP nanoparticles was 3.4 a.u. (25 mg), which is the highest achieved so far in one manufacturing cycle using the following conditions: monomer concentration 1.8% to 3.25%, irradiation time 2.5 to 2.6 min, and the identical temperature of irradiation and low-affinity wash at 10°C. These results clearly prove the validity of the DOE approach used here for the optimization of MIP nanoparticle yield. Moreover, it was shown the properties of the particles synthesized at optimum conditions had binding affinity similar to monoclonal antibodies.

Future works may also consider using different parameters (for example, cross-linker concentration and type of solvent) for the optimization of nanoMIP yield or binding characteristics. Finally, in reference with other works summarized in review [13], this study has shown that DOE can be used as a rational approach to MIP optimization. Thus, this approach can be used in the future for up-scaling of MIP production for commercial application.

## Abbreviations

APTMS: 3-aminopropyltrimethyloxysilane; CCF: central composite on face; Cmon: concentration of monomer; DOE: design of experiments; MIPs: molecularly imprinted polymers; nanoMIPs: molecularly imprinted polymer nanoparticles; OVAT: one-variable-at-the-time; PBS: phosphate buffered saline; RSM: response surface methodology; T_Laf: temperature of low affinity waste; Temp: temperature of irradiation; Tuv: irradiation time.

## Competing interests

The authors declare that they have no competing interests.

## Authors’ contributions

KM carried out the experimental design and took part in the synthesis of MIP nanoparticles, KK participated in sequence alignment and drafted the manuscript. AG carried out the nanoMIP yield assay. AP participated in the preparation of template-derivatized glass beads and took part in synthesis of MIP nanoparticles. SP participated in the design of the study and performed the data analysis. All authors read and approved the final manuscript.

## References

[B1] Piletsky S Turner A Molecular Imprinting of Polymers 2006 Georgetown: Landes Bioscience

[B2] Moreno-Bondi MC Benito-Peña ME Urraca JL Orellana G Immuno-like assays and biomimetic microchips Top Curr Chem 2012 9 111 164 22415415 10.1007/128_2010_94

[B3] Chen LX Xu SF Li JH Recent advances in molecular imprinting technology: current status, challenges and highlighted applications Chem Soc Rev 2011 9 2922 2942 10.1039/c0cs00084a 21359355

[B4] Muzyka K Piletsky S Rozhitskii M Alvarez-Lorenzo C Molecularly imprinted polymer-based voltammetric sensors Molecularly Imprinted Polymers: a Handbook for Academia and Industry 2013 UK: iSmithers 197 228

[B5] Poma A Guerreiro A Whitcombe MJ Piletska EV Turner APF Piletsky SA Solid-phase synthesis of molecularly imprinted polymer nanoparticles with a reusable template–“plastic antibodies” Adv Func Mater 2013 9 2821 2827 10.1002/adfm.201202397 PMC4746745 26869870

[B6] Subrahmanyam S Karim K Piletsky SA Piletsky SA, Whitcombe MJ Computational approaches in the design of synthetic receptors Designing Receptors for the Next Generation of Biosensors 2013 Berlin Heidelberg: Springer 134 166

[B7] Piletska EV Guerreiro AR Whitcombe MJ Piletsky SA Influence of the polymerization conditions on the performance of molecularly imprinted polymers Macromolecules 2009 9 4921 4928 10.1021/ma900432z

[B8] Leardi R Experimental design in chemistry: a tutorial Anal Chim Acta 2009 9 161 172 10.1016/j.aca.2009.06.015 19786177

[B9] Verma A Hartonen K Riekkola M Optimisation of supercritical fluid extraction of indole alkaloids from Catharanthus roseus using experimental design methodology - comparison with other extraction techniques Phytochem Anal 2008 9 52 63 10.1002/pca.1015 17654538

[B10] Lin J Su M Wang X Qiu Y Li H Hao J Yang H Zhou M Yan C Jia W Multiparametric analysis of amino acids and organic acids in rat brain tissues using GC/MS J Separation Science 2008 9 2831 2838 10.1002/jssc.200800232 18668507

[B11] Kempe H Kempe M Novel methods for the synthesis of molecularly imprinted polymer bead libraries Macromolecules. Rapid Commun 2004 9 315 320 10.1002/marc.200300189

[B12] Mijangos I Villoslada FN Guerreiro A Piletska EV Chianella I Karim K Turner APF Piletsky SA Influence of initiator and different polymerisation conditions on performance of molecularly imprinted polymers Biosen Bioelectron 2006 9 381 387 10.1016/j.bios.2006.05.012 16782322

[B13] Nicholls IA Andersson HS Golker K Henschel H Karlsson BCG Olsson GD Wikman S Rational design of biomimetic molecularly imprinted materials: theoretical and computational strategies for guiding nanoscale structured polymer development Anal Bioanal Chem 2011 9 1771 1786 10.1007/s00216-011-4935-1 21475943

